# Assistive Technology to Improve Collaboration in Children with ASD: State-of-the-Art and Future Challenges in the Smart Products Sector

**DOI:** 10.3390/s22218321

**Published:** 2022-10-30

**Authors:** Raquel Cañete, Estela Peralta

**Affiliations:** Escuela Politécnica Superior, Universidad de Sevilla, 41011 Sevilla, Spain

**Keywords:** assistive technology, multisensory integration, smart products, interactive products, autism spectrum disorder, social skills, collaboration, cognitive disabilities, intellectual disabilities, training for independent living

## Abstract

Within the field of products for autism spectrum disorder, one of the main research areas is focused on the development of assistive technology. Mid and high-tech products integrate interactive and smart functions with multisensory reinforcements, making the user experience more intuitive, adaptable, and dynamic. These products have a very significant impact on improving the skills of children with autism, including collaboration and social skills, which are essential for the integration of these children into society and, therefore, their well-being. This work carried out an exhaustive analysis of the scientific literature, as well as market research and trends, and patent analysis to explore the state-of-the-art of assistive technology and smart products for children with ASD, specifically those aimed at improving social and communication skills. The results show a reduced availability of products that act as facilitators of the special needs of children with ASD, which is even more evident for products aimed at improving collaboration skills. Products that allow the participation of several users simultaneously through multi-user interfaces are required. On top of this, the trend toward virtual environments is leading to a loss of material aspects in the design that are essential for the development of these children.

## 1. Introduction

Autism Spectrum Disorder, hereinafter ASD, is a neurological disorder; it encompasses different types of needs, making it difficult to develop product and service solutions that are adaptable to everyone [1]. Autism is characterized by two main signs: (i) restrictive and repetitive patterns of interests, behavior, or activities, and (ii) persistent communication and social interaction difficulties. The areas where they have the most difficulty are social interaction, imagination, and communication. People with ASD tend to isolate themselves and show no interest in others. This is the aspect that most affects their well-being [2]. Their lack of social awareness, as well as their refusal to share, makes collaboration with other children more difficult. These children have difficulties adapting [3,4,5] and developing in collaborative games given their inflexibility and their difficulty predicting responses and identifying the emotions of their partner.

These difficulties have an impact on the deficiencies in interaction, communication, and social imagination [6], essential skills for child independence [5]. In addition to this, they can cause anxiety in children [3,7]. Other indirect impacts such as bullying, social rejection, and school dropout also arise [5]. Therefore, for these children, it is important to acquire collaborative skills that improve their behavior in the social environment in which they live [3].

Due to this challenge, the importance of cooperative play in children with ASD is emphasized. Learning to work together in reciprocity and maintaining joint attention with shared activities and goals achieved increases trust in the partner and improves conflict management, monitoring of norms, understanding of common interests and objectives, awareness and social interaction, decision-making, making requests, acceptance of the results and adaptation of behavior to the environment, among others [6,8,9,10]. In this context, assistive technology (AT) plays an important role in the development of collaborative activities. AT can be used as an intermediary element that encourages and enhances collaboration in the development of tasks. To optimize its usability, it must comply with universal design principles as a requirement, integrating physical, cognitive, and sensory accessibility. Currently, the supply–demand relationship is inadequate; the variety of quality products specialized in ASD is scarce. Furthermore, those marketed with the correct value for money are not affordable for the entire population. Lastly, in most cases, there are solutions available on the market that cover the broad set of needs related to communication, social interaction, or behavior. 

This work carries out an exhaustive study of the scientific literature, as well as a market study and patents related to assistive technology specialized in ASD. The aim of this research is to explore the state-of-the-art of assistive technology, including interactive and smart products aimed at improving the skills of children with ASD; specifically, the scope includes those that help in the development of social and communication skills through collaboration, with the aim of: (1) Analyzing the current research effort in this line of work by the scientific community, (2) identifying the current supply-demand situation of assistive technology in terms of usability, affordability, and availability of products; and (3) defining the lines of work and main challenges in the development of AT for the improvement of the quality of life of children with ASD, with the aim of properly guiding the effort of Research & Development activities.

To do this, the article is structured as follows: Section 2 describes the methods used in the review. Section 3 develops the results of the review, structured in three phases: analysis of the scientific literature, analysis of patents, and analysis of commercial solutions. Section 4 includes a discussion of the results that identify future work lines and the main challenges related to the development of AT to improve the quality of life of children with ASD. Lastly, Section 5 discusses the main conclusions of the study. 

## 2. Methods

The review presented in this article focused on different contexts: (I) existing solutions focused on databases of scientific sources, (II) patented solutions and intellectual property (IP), and (III) commercial product materials in web sources.

### 2.1. Scientific Literature Review

The main objective of this article is to provide an overview of the current state of assistive technology (including interactive and smart products) that aims to improve the skills of children with ASD. Specifically, devices that help develop social and communication skills were considered.

Literature review was conducted for original research papers. The literature search was carried out between 7 January 2022 and 14 April 2022.

For this search, combinations of keywords were used such as: “COLLABORATION” “METHODOLOGY” “ASD” OR “TOOLS” “COLLABORATION” “CHILDREN” “AUTISM” OR “SMART” “PRODUCTS” “COLLABORATION” “AUTISM” OR “COLLABORATIVE” “GAME” “AUTISM”; as well as keywords such as: “ROBOTS” “AUTISM” OR “ASSISTIVE TECHNOLOGY” “AUTISM” OR “INTERACTIVE PRODUCTS” “AUTISM” OR “DESIGN GUIDELINES FOR AUTISM” OR “COGNITIVE DISABILITIES” OR “INTELLECTUAL DISABILITIES”. 

**Figure 1:**
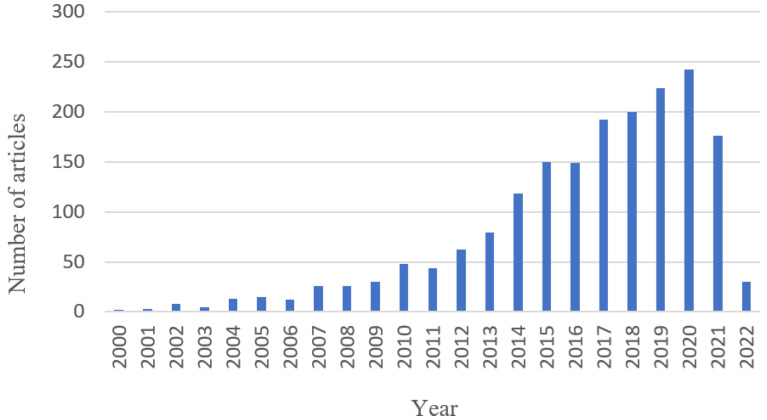
Article record 2000–2022 in Web of Knowledge (WOK).

Records from the Institute for Scientific Information (ISI) Web of Knowledge (WOK) were restricted to the years 2000 to 2022. A total of 1852 records were identified (Figure 1).

In the end, 328 records were included for analysis, integrating scientific articles, books, conferences, theses, and generics. Table 1 shows the statistics of the different types of sources. 

These records were divided into different categories:

1. Article Reviews: state-of-the-art of medium/high assistive technology tools for children with ASD.
2. Autism (basis): articles dedicated to autism spectrum disorder, focusing on characteristics, symptoms, and needs to be covered with the products.
3. Autism (models and therapies): articles focused on methodologies and therapies focused on the development of skills in children with ASD. This category includes papers that discuss the theoretical framework or propose new therapies.
4. Techniques and tools (basis): articles that describe techniques and tools used to improve the skills of children with ASD.
5. Techniques and tools (case studies): works whose objective is the development of assistive technology that is tested on users.
6. Design Methodologies and Guidelines: works focused on improving the product design process; methodologies, methods, good practices, or assistive technology design guides are proposed. This category is further subdivided into: (1) design methods specialized in ASD, (2) design methods for typical development; and (3) participatory design.
7. Play and Toys: articles related to characteristics and forms of play for children with typical development and ASD, as well as work focused on the study of characteristics and trends of toys for both groups.
8. Interactive and Smart Design: development of interaction design theory.
9. COVID19 and Autism: analysis of the impacts of the pandemic and how it has generated new needs for assistive technology products.

This last category is included given the number of articles dedicated to the relationship between COVID-19 and its impact on children with ASD during the last two years (2020–2022). This selection of articles is considered relevant to the study, as the extreme circumstances of this pandemic have highlighted the importance of assistive technology in the domestic context of children with ASD and their families. Furthermore, the new work and academic models that have been implemented after the pandemic (such as telework or flexible hours in the work environment) have created the need for new strategies to be able to balance work life with family life, thus emerging the need for new assistive technology products focused on these new contexts. 

Table 2 shows the different categories, as well as the number of records in each category, and the range of dates that are analyzed according to the records found.

Of these records, 203 are papers published in scientific journals included in the journal Citation Reports. Table 3 summarizes the nine main journals for the articles included in this work, classified according to the frequency of publication in the following order: “*Journal of Autism and Developmental Disorders*”, indexed in the category of psychology and development within the first quartile (Q1); “*International Journal of Social Robotics*”, is a technical journal indexed in the category of robotics (Q1); and “*Research in Autism Spectrum Disorders*”, “*Computers in Human Behavior*”, “*Research in Developmental Disabilities*”, “*Autism*” and “*Autism Research*”, indexed in psychology and education in the first quartile (Q1). The journals “*Sensors*” (engineering and chemistry categories in the second quartile Q2) and “*ACM Transactions on Accessible Computing*” (computer science category in the fourth quartile Q4) are technical journals. 

On the other hand, the geographical areas represented in the affiliations of the 319 selected resources, which covered 50 countries, were analyzed. Table 4 shows the most representative. Among them, the United States stands out, representing 31.40% of the resources, followed by the United Kingdom (12.20%) and Spain (9.76%).

### 2.2. Patent Analysis

A patent search and study were conducted using the “WIPO Patent Scope” database between 28 March and 3 April 2022. This search was aimed at collecting patents for smart and interactive assistive technology products aimed at improving the collaboration and social skills of children with ASD. These products were divided into three groups: Virtual Environments, Robots, and Toys. For this search, results were found between April 2007 and March 2022. Table 5 details the searches performed, as well as the number of results obtained. In the end, 55 patents were selected to perform the analysis.

A sample of 55 patents was selected to perform the analysis. The IPC (International Patent Classification Code) was used to provide relevant information, such as the main areas and fields of the patents, the location of the target area of the applications in the patent consortium, and the details for the evolutionary potential analysis. Although a new code system called CPC (Cooperative Patent Classification) is currently being implemented, it was not used because some of the selected patents were not up to date during the search period. For the analysis and visualization of the results based on IPC codes, the PatentInspiration software was used.

### 2.3. Analysis of Commercial Assistive Technology Products for Collaboration in Children with ASD 

AT solutions are marketed primarily outside of information published in research sources, such as scientific journals or conference articles. Therefore, this commercial search strategy was carried out using Google. The search for commercial solutions was carried out between 18 March 2022, and 25 March 2022, to find solutions marketed on the global market, with descriptions available in English.

In the first phase of this section, a search was carried out for companies dedicated to the development of assistive technology for children with ASD. These companies were divided into two groups: (i) companies dedicated to the medium-high assistive technology sector, and (ii) companies in the toy sector. This last category was included because many of the products specialized in ASD currently available on the market are intended for children, so the AT is configured as a toy to enhance the child’s attention and improve the acceptability of tasks. The categorization of products from these companies was studied, as well as the offer available for categories related to collaboration.

As a second phase of the commercial analysis, a search was carried out for popular AT products aimed at improving collaboration in children with ASD. About 30 products were selected and divided into three categories: (i) mobile applications and virtual environments, (ii) robots, and (iii) toys. Then, these products were analyzed within the collaboration categories.

## 3. Results

This section describes the results of the analysis of assistive technology and interactive and intelligent products, focused on the development of communication skills and social skills of children with ASD, with the objective of analyzing the current situation of scientific literature, patents, and commercialized products. 

### 3.1. Literature Review

**Figure 2:**
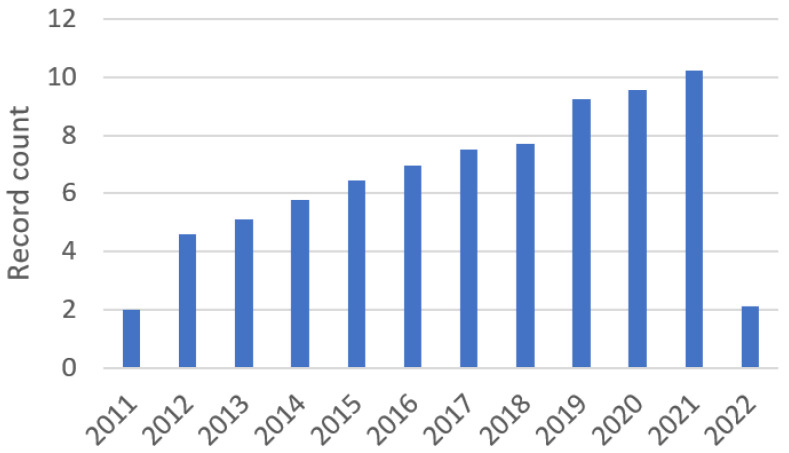
Trend of scientific studies related to autism (2011–2022).

Currently, 1 in 44 children is diagnosed with ASD, which corresponds to approximately 2.27% of the population [11]. The increase in the number of diagnoses, together with the fact that their origin is unclear in 90% of cases [12], has led to an increase in the number of studies related to autism in recent years, being one of the most studied pathologies in the scientific literature. Figure 2 presents the trend of scientific studies related to autism according to ISI WOK (“AUTISM”), with a total of 75,291 resources in the last 20 years (2012–2022).

Specifically, research related to assistive technology specialized in ASD has increased in the last decade. These studies range from theoretical and analytical studies to technological developments with user validation. For the present work, resources related to the development of interactive and/or intelligent products for the improvement of social and collaborative skills of children with ASD are considered.

**Figure 3:**
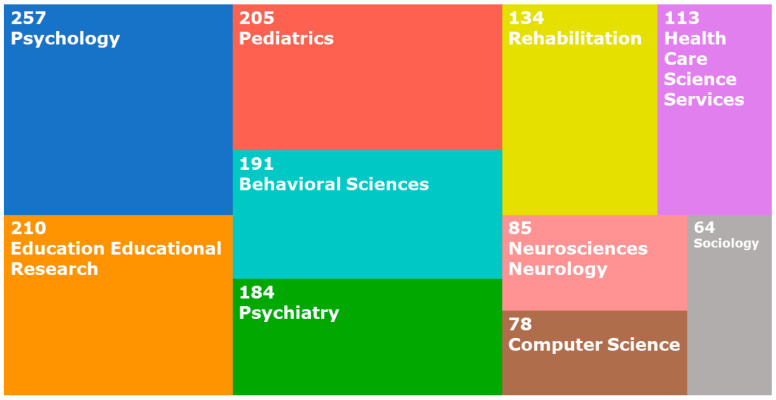
Most frequent research areas in the bibliographic study.

The ten most frequent research areas covered by ISI WOK results in the search “COLLABORATION” AND “CHILDREN” AND “AUTISM”, include 590 results, which are classified in Figure 3.

Within this classification, the 213 scientific articles related to the objective of the review are indexed in the categories of Journal Citation Report shown in Table 6.

By analyzing Figure 3 and Table 6, it can be seen that most of the resources focused on studying the collaboration and social skills of children with ASD, as well as the therapies and tools used to improve them, are found more frequently in the fields of psychology and education. Research is less often related to the technical disciplines of engineering, computer science, or design.

**Figure 4:**
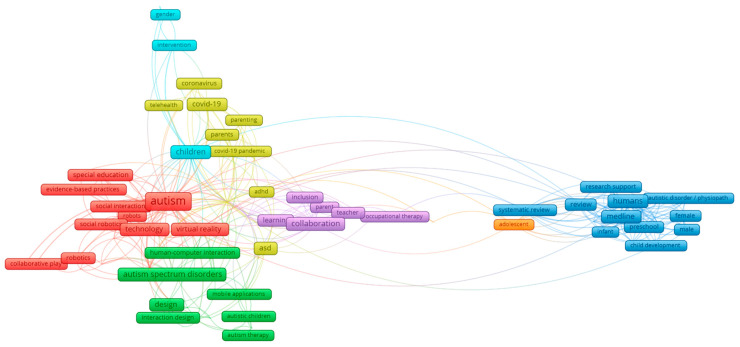
Keyword analysis for the bibliographic study.

The bibliographic search was analyzed by clustering. The VOS Viewer SW tool was used, which provides visually indicative clusters. Of the 328 resources analyzed, 1179 keywords (659 different) were obtained for which full counting was used. Figure 4 shows the 7 clusters obtained with 87 elements (keywords) and 371 links between keywords (the minimum number of occurrences of a keyword was two):

- Green cluster: groups words related to autism spectrum disorder and intervention and therapy with assistive technology tools.
- Blue cluster: collects words related to the epidemiology of autism spectrum disorder and the development of these children.
- Red cluster: groups the words related to autism and its needs for social inclusion.
- Yellow cluster: groups the words related to the COVID-19 pandemic and autism.
- Purple cluster: collects words related to autism spectrum disorder and collaboration between children, parents, and professionals.
- Light blue cluster: refers to words related to toys, autism, and stereotypes.
- Orange cluster: collects words related to the social participation of adolescents.

As established in Section 2 “Methods”, to structure the review, the set of selected works was classified into nine categories: Article Reviews, Autism (basis), Autism (Models and Therapies), Techniques and Tools (basis), Techniques and Tools (case studies), Design Methodologies and Guidelines, Play and Toys, Interactive and Smart Design, and COVID19 and Autism. Of the 328 resources analyzed, 248 are specifically dedicated to autism, the rest being resources focused on special needs in general (28), and design articles based on typical development (52). Table 7 shows the number of resources specifically focused on autism for each category.

Within these 248 resources focused on autism, 122 are specifically dedicated to collaboration and social skills. These are classified in Table 8. As can be seen, the largest number of articles focused on collaboration and social skills is in the categories of Techniques and Tools (Case Studies with 34.5% and Basis with 18%). Within these categories, the resources found in “Techniques and Tools (basis)” are primarily focused on exploring the potential of assistive technology in social and collaboration skills of children with autism, including and comparing robots, virtual environments, interactive products, and others. These records also discuss the benefits these systems could have in education and classrooms, arguing that the use of these types of products could have very positive effects on the collaborative skills of these children [13,14,15,16,17]. On the other hand, in the category of Techniques and Tools (Case Studies) records are dedicated to testing several AT solutions with children with autism. These solutions include apps, virtual environments, interactive products, robots, and others. Most of the resources in this category were focused on virtual environments. This will be further analyzed in this section. Although the category of Design Methodologies and Guidelines is the second most significant category with 26.4%, most of the studies found in this category (30/32) conceptually describe the importance of participatory design with children with ASD, families, and professionals to obtain efficient AT solutions, however, they do not develop concrete design methods or design processes. On the other hand, the other two resources that do propose specific methods are focused on virtual environments, but no specific methods have been found for other types of high-tech products, such as robots or smart products, nor have they been found for toys. Thus, there is a clear shortage of design methods and tools dedicated to the development of AT products that improve the collaboration and social skills of children with ASD. It is also worth noting the low frequency of publications dealing with collaboration in the category of Play and Toys, and the nonexistence of studies in the categories of Interactive and Smart Design and COVID-19 and Autism.

After analyzing in detail this classification, it should be noted that in the first category (Article Reviews), 15 scientific articles and 1 conference article dedicated to the study of the state-of-the-art of interactive and intelligent products were found (see Table 7). However, several of these reviews are of general scope, that is, they are not oriented to any specific product or need [18,19,20]. Other studies look at a particular type of assistive technology, such as virtual environments [21,22,23], or robots [24,25,26,27,28]. Likewise, it is worth noting that the only two review studies whose scope is “collaboration” (see Table 8) are developed by Baykal et al., where the development of social and communication skills in populations with special needs is evaluated [29], and Silva-Calpa et al., with the focus on virtual environments specialized in autism [10]. This work has not found studies in this category that review the current state of development of AT specializing in collaboration and social skills for ASD.

The concept of assistive technology developed in these investigations includes any device, software, or equipment that helps overcome certain challenges that require assistance to perform activities of daily living independently. Specifically, a subclassification can be identified according to the scope of the AT:

- Low-tech products: traditional tools and methods that use non-interactive products or do not use energy.
- Medium/high technology: with electronic and computerized elements that improve efficiency, speed, and accessibility.

Works with this scope belonged to the following categories: 4. Techniques and tools (basis), 5. Techniques and tools (case studies), and 6. Design Methodologies and Guidelines. Of the 248 resources found, 157 are specialized in ASD: 44 develop AT in general (treat both low and medium/high technology) (28.76%), 3 Low-tech (1.96%), and 106 Mid/high tech (69.28%). These results show the trends of the research effort in the last decade. This situation may be due to the benefits of mid/high-tech AT compared to other types of products [30,31,32]. 

**Figure 5:**
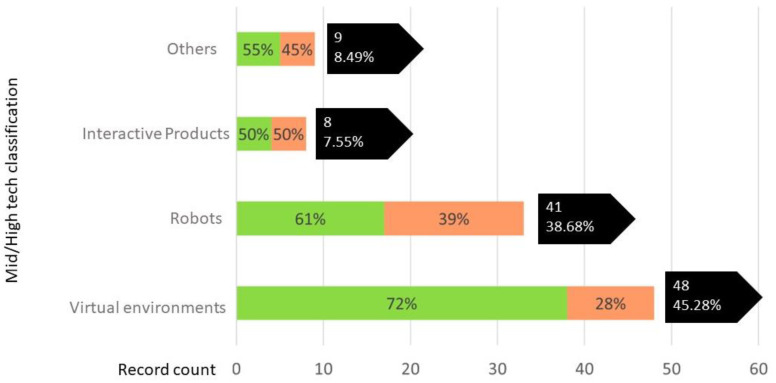
Mid/high tech product classification for ASD.

The resources included in the mid-high tech category were classified into: (1) Virtual environments (mobile applications, video games, virtual and augmented reality environments...), (2) Robots (robots intended for therapy of children with ASD), (3) Interactive products (Toys, wearables), (4) other tools in product form that do not belong to any of the above categories. Figure 5 shows the distribution of the research effort (%) of a total of 106 papers; those works that include in their scope the development of collaboration and social skills in the design of the product are indicated in green. 

After this analysis and from Figure 5 it can be concluded that most of the assistive technology developed for children with ASD belongs to the category of virtual environments (45.3%), especially those focused on improving the collaboration and social skills of children with ASD (72%). These applications and virtual environments can be very beneficial as they offer great flexibility in content and customization. Highlight the following studies, which demonstrate the potential of such applications in the therapy of children with ASD [2,4,6,14,15,16,17,33,34,35,36]. However, these types of tools lack tangibility and materiality, aspects especially important for the intuitiveness and stimulation of these users [37,38]. Therefore, it is essential that the research effort is not limited to the study of exclusively virtual solutions, but combines traditional strategies, methods, and materials with new technologies to develop more complete products (without losing their tangibility and materiality), which are flexible and adaptable to the characteristics of the user.

Second, research focuses on the development of “social robots” (38.7%). Analyzing the publication dates of these works, it is concluded that it is a full development research line, where most of the works aim to demonstrate the benefits offered by the use of robots in therapy for children with ASD [13,24,30,31,39,40,41,42,43]. 

The category “smart and interactive products” is the least developed. This category integrates all kinds of interactive and smart products that do not belong to the previous categories, including the AT configured as a toy. This work bases the understanding of toys on Sicart’s definition: “a thing at the service of playing and the playful, and that is why it is an instrument for self-expression, self-knowledge, and exploration” [44]. This category integrates toys that, in addition to serving as play instruments, help develop certain skills in children with ASD, both physical and cognitive. These differ from social robots in that they are focused on children’s play and fun. However, these functions can be complemented by the development of certain skills. In addition to this, they usually integrate a lower technology, which causes them to usually have specific functions and do not present multifunctionality. This AT is generally better value for money and, therefore, affordable and available for family settings. It should be noted that 100% of articles that develop toys specialized in ASD conceptually identify the importance of collaborative play, but no research obtains representative results related to functional and non-functional requirements to be included in the products, as well as preferences in terms of usability (efficiency, effectiveness, and satisfaction in the interaction between children and ASD-product). 

It should be noted that “play” is an “intrinsic activity, playing for the sake of playing, which arises as something spontaneous and voluntary, not out of obligation, and includes an element of pleasure, because it is done to have fun” [9]. Collaborative play can intervene not only in the playful field, but also in the sensory, motor, and cognitive. It is important to create communicative environments in games and play, to give the child the possibility to express desires and needs and develop their communicative skills. It is essential to create situations that require him to ask for objects, comment, protest, etc. These strategies introduce the user to a cause-and-effect process, improving attention, tracking visual stimuli, and recording instructions. In cooperative play for children with ASD, the verbal description of each activity during the process is very important. For this purpose, the need to introduce adapted reinforcements (visual, auditory, tactile…) is created [3]. It may also be beneficial to allow the child to choose his own materials and adapt the game according to his preferences. In addition to this, it is important that the game has a clear context [4] and resembles real-life interactions [3]. Similarly, during cooperative play, it is possible to observe parameters such as the level of correspondence, the degree of alternation between playmates, and the degree of variability in responses, which are properties of reciprocal interaction that can indicate the level of social development [3]. The lack of research that develops new knowledge related to collaborative play through interactive and smart products and AT to improve the social and collaborative skills of children with ASD makes it a line of work of interest, given the importance of these skills in the quality of life and independence of these users. 

From the last category “COVID and Autism” we could deduce that COVID-19 has imposed changes on everyone’s social life and the job market that could be sustained and made permanent, causing physical and mental challenges to the population. As a result of this new reality, improved family and work conciliation will be necessary, which will be particularly difficult for families with children with autism [45,46,47,48]. Thus, it is further underlined that there is a need for solutions to assist parents in balancing employment and the care of these children. One of the main disadvantages of these products is their affordability; due to their high price, they are mostly used as professional tools in therapy, but cannot be used in a domestic context. Therefore, from this category, it can be deduced that there is a need for products that are affordable, aimed at a domestic context and that can be used at home by parents and children.

### 3.2. Patent Applications of Assistive Technology

This section shows the results of the analysis of assistive technology patent applications to improve the social and collaborative skills of children with ASD. 

**Figure 6:**
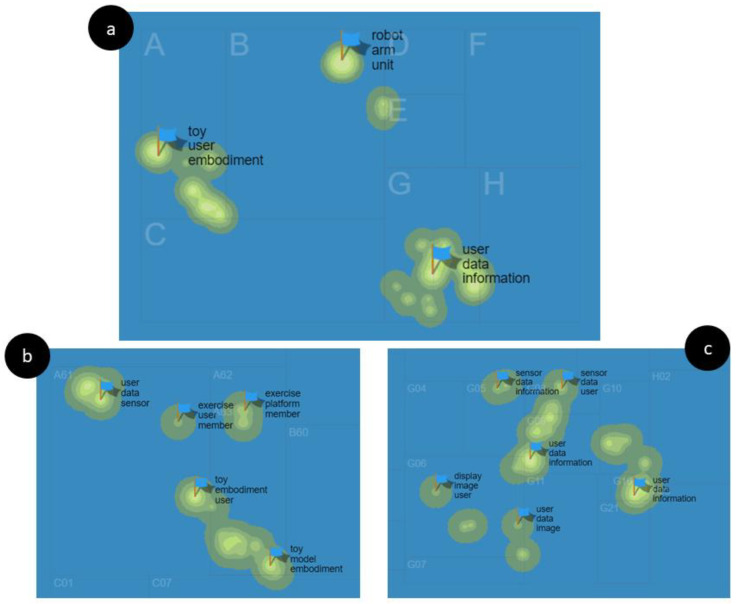
IPC Code Map for patent sample: (**a**) areas of scope of the selected sample (**b**) (1) A-HUMAN NECESSITIES areas, and (**c**) (2) G-PHYSICS areas.

The IPC Code Map (Figure 6a) represents the areas of scope of the 55 patents of the selected sample, the most common being: (1) A-HUMAN NECESSITIES, more specifically in A6-HEALTH; AMUSEMENT (Figure 6b) and (2) G-PHYSICS, specifically in G0 and G1-INSTRUMENTS (Figure 6c).

**Figure 7:**
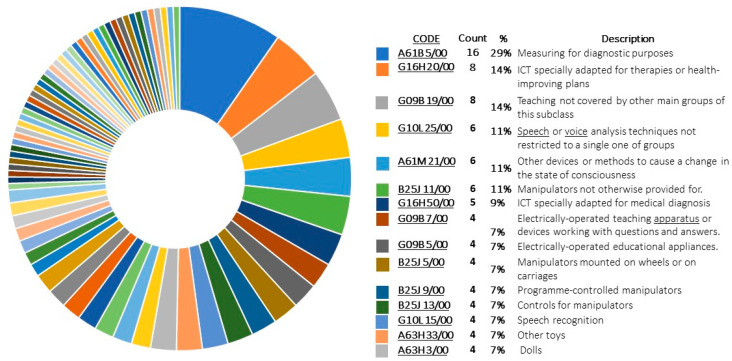
Most common IPC codes in the patent sample.

Figure 7 shows the 10 most frequent IPC codes in the patent sample, the most representative being: (1) A61B 5/00 Measuring for diagnostic purposes (29.09%); (2) G16H 20/00 ICT specially adapted for therapies or health-improving plans (14.55%), (3) G09B 19/00 Teaching not covered by other main groups of this (14.55%). Code (4) G10L 25/00 Speech or voice analysis techniques not restricted to only one of the groups (10.91%), (5) A61M 21/00 Other devices or methods to cause a change in the state of consciousness (10.91%), and (6) B25J 11/00 Manipulators not otherwise provided for (10.91%).

By grouping similar IPCs, it is possible to create clusters of patents organized into sections and technological areas. In the case of the sample analyzed, the following sections were obtained: (1) Electrical Engineering, (2) Instruments, (3) Mechanical Engineering, and (4) Other Fields, whose technological areas are shown in Table 9.

**Figure 8:**
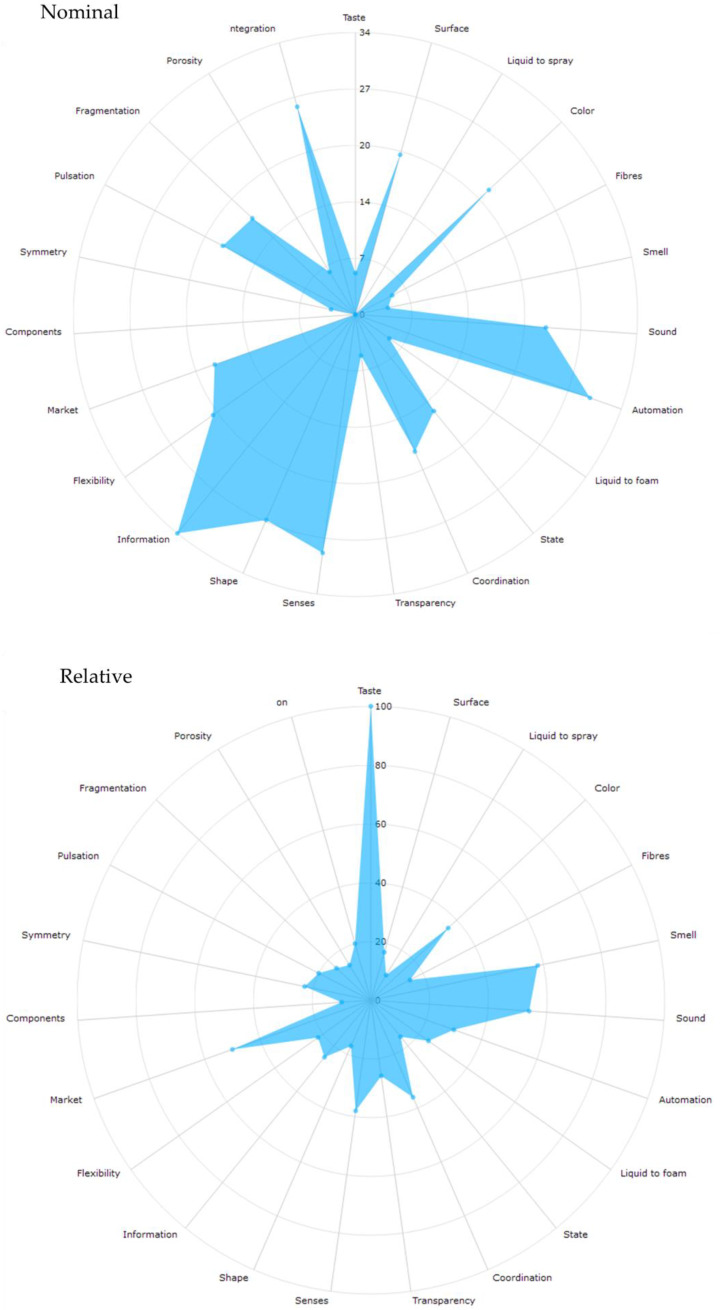
Evolutionary potential analysis graph based on the patent pool.

From patent analysis, it is possible to obtain particularly interesting information about the innovation potential of the sector. For this purpose, the “evolutionary potential analysis” was used; this evaluation shows a list of properties as well as their relevance within a selected patent sample. Likewise, it establishes a quantification indicator, called Nominal value (Figure 8a), and a weighting indicator, called Relative value (Figure 8b). 

First, the nominal value indicates the number of patents in the sample that deal with a particular property; it analyzes the total number of patents that prioritize a property within the sample. In the selected sample, the priority properties are Information and Automation along with other less frequent properties such as Taste, Porosity, or Components. On the other hand, the relative analysis shows the use of each property in the total number of patents in the sample; this indicator identifies the innovation potential of each property, which is represented in the white space of the graph. In the selected sample, the Taste, Smell, or Market properties have great innovation potential.

The search, selection, and analysis of patents were divided into three types of products: “Virtual environments” (Table 10), “Robots” (Table 11), and “Toys” (Table 12).

Within the “Virtual Environments” group, different types of environments are identified. Several patents develop therapy devices, such as those of [49] that focus on the recognition and expression of emotions through videos; and [50] a wearable that, by analyzing the child’s eye contact, verbal interaction with the caregiver, and repetitive movements of the head and body, performs an analysis and monitoring of the evolution of the child. Likewise, patents focused on the analysis of children’s social skills are common with the aim of identifying and detecting signs and symptoms of autism; making use of sound analysis of the environment with microphones and recorders [51,52] or using motion detection cameras [53]. Highlight the proposal of [54] with a comprehensive rehabilitation training system based on 3D printing technology, with different modules to train different skills.

Regarding virtual platforms, the proposals aim to develop language and communication based on image exchange systems (PECS) [55], develop concrete skills through reward systems [56,57,58], or improve skills through gamification (games) [59]. Some virtual environments allow the user to create simulation units that are used to present images of avatars or characters, social scenes, and situations and stories that improve collaboration [60].

Specifically, of the virtual environments intended for collaboration, those that use virtual reality systems to present social situations stand out [61,62,63,64,65]. In addition to this, there are some proposals that can be linked to other types of product, such as robots [66]. 

**Table 10:** Sample of assistive technology patents in the form of virtual environments.

S/N	Reference	Title	Publication Number	Patent Issue Date	Patent Right Holder	Country
1	[49]	Intelligent assistive device for Tamilnadu autistic children	IN202241009551 A	11 March 2022	Dr. A. Benjamin Joseph Mr. D. Prakash Ms. Lourdu Jennifer J.R Mr. R. Thirumurugan S. A. Engineering College	India
2	[55]	Generative language training using electronic display	US20190130780 A1	19 December 2018	Oliver Wendt	USA
3	[50]	Systems, environment, and methods for emotional recognition and social interaction coaching	US20190015033 B2	17 Jenuary 2020	Nedim T. Sahin	USA
4	[56]	Electronic platform aiding persons having autism	IN201941032153 A	12/02/2021	Meenakshi Kumar Kotra Jerry Thomas Naga Mohan Kumar Rajasekhar Reddy Jonnalagadda	India
5	[59]	Child development platform	US20160027323 A1	28 February 2016	Conlan MA Thanh Tran	USA
6	[51]	System and method for expressive language, developmental disorder, and emotion assessment	WO2010085681	29 July 2010	Xu, Dongxin, D. Paul, Terrance, D.	World
7	[57]	Personalized digital therapy methods and devices	WO2020198065	20 March 2020	Vaughan, Brent Taraman, Sharief Khalil Abbas, Abdel Halim	World
8	[52]	Systems and methods for expressive language, developmental disorder, and emotion assessment, and contextual feedback	US20160351074 B2	1 December 2016	Terrance D. Paul	USA
9	[67]	Virtual Reality Medical Application System	US20190366030 A1	5 December 2019	Huan Giap Garland Wong	USA
10	[54]	Multi-sensory training system based on 3d printing technology	CN108744220 B	6 November 2018	Gu Jingxin Yang Shangqing	China
11	[66]	Robot-mediated tele-rehabilitation system for autism therapy	MYPI 2017703633 A	27 March 2019	Hanafiah Bin Yussof	Malaysia
12	[65]	Autism fusion training system based on VR technology	CN210433827 U	20 March 2019	Cai Zhihua	China
13	[68]	Mind controlled gaming for the differently abled	IN201841016343	11 May 2018	K. Palanikumar B. Sreedevi P. Navaneeth H. Akshay M. Nirmalraj S. Athreya	India
14	[60]	Autism intervention system integrated with real character image	CN108665555 A	16 October 2018	Liu Leyuan Chen Liangying Gui Wenting Zhang Kun Liu Sannyuya Yang Zongkai Xu Ruyi Peng Shixin	China
15	[61]	System and method for autism children to practise social skills by using virtual reality technology	IN202021008757 A	1 March 2020	Bhaskar Vijay Ajgaonkar	China
16	[63]	Design of personalized virtual home to teach fire safety skills for autism spectrum disorder	IN202141006253 A	19 February 2021	Nithya Shree T	India
17	[64]	Autism training system, method and device based on virtual reality technology	CN111009318 A	14 April 2020	Zhai Guangtao Fang Yi Fan Lei	China
18	[62]	Social communication function training method for children with autism spectrum disorder	CN113284625 A	28 August 2021	Li Jia	China
19	[58]	An autism spectrum disorder children cooperation ability intervention system and method	CN109599162	9 April 2019	Yu Dongchuan Miao Jia Zhang Lei	China
20	[53]	Web server based 24/7 care management system for better quality of life to alzheimer, dementia, autistic and assisted living people using artificial intelligent based smart devices	US20180253954 B2	30 March 2021	Shiv Prakash Verma	USA

Regarding robot development, designs include multiple sensors that allow for detecting different aspects of human–machine interaction (HMI); such as vision cameras and voice modules, useful for automatically recognizing the patient’s emotion and deducing the meaning of language according to their facial expression [69], or for recognizing emotions and creating a conversation and interaction according to collected data [70]; displacement sensors, sound pickup, facial expression recognition, behavior recording, voice output for visualization of emotions [71]; or user-programmable processors [72], in order to instruct the robot to perform therapeutic interactive movements, gestures, and audiovisual signals. They also combine image acquisition devices with sound collectors and players [73], or motion sensors to capture motor reactions of users [74,75]. These robots that monitor information also allow in many cases to adapt and personalize the interaction in a way that improves and makes more comfortable the child’s interaction with the ASD-Robot. In addition to this, they also serve to monitor and follow therapy progress [76,77,78].

With respect to the functional scope of robots, in general, the most developed categories are the recognition of emotions and facial expressions [79]. It is worth highlighting the proposal of [80], an emotionally expressive robot that participates in sensory experiences by reacting to stimuli that simulate typical everyday experiences, such as uncontrolled sounds, light, or tactile contact with different textures. The goal is to teach children with ASD to express their emotions toward different sensory experiences; or the robot developed by [81], which uses the automation of behavioral analysis to provide treatment and evaluation of emotional communication and social skills for children with autism. Proposals for the development of language and communication are also common, [82], as well as for improving imitation skills, which are of great importance in social skills [83] or in improving eye contact [84]. Robot patents that are intended for the domestic context allow to work on different skills of daily life [85] or identifying objects (using for example, RFID tags next to a verbal or gestural response) [86]. In addition to this, robots can be combined with other types of products, such as virtual environments, such as holograms [87].

**Table 11:** Sample of assistive technology patents in the form of robots.

S/N	Reference	Title	Publication Number	Patent Issue Date	Patent Right Holder	Country
21	[69]	Robot for adjuvant therapy of infantile autism	CN107283389 A	16 March 2021	Li Jinglong	China
22	[71]	Auxiliary communication device for children with autism	CN107307865 A	3 November 2017	Dong Rongqin	China
23	[85]	Intelligent Home Robot for Treating Autism	CN108536179 A	17 September 2018	Shi Azhen	China
24	[86]	An Interactive Humanoid Robot Using RFID Tagged Objects	GB2552981 B	21 February 2018	Kerstin Dautenhahn, Ben Robins, Luke Wood	UK
25	[82]	Interactive Systems Employing Robotic Companions	US20090055019 B2	26 February 2009	Stiehl Walter Dan, Breazeal Cynthia, Lee Jun Ki, Maymin Allan Z, Knight Heather, Toscano Robert L., Cheung Iris M	USA
26	[72]	Therapeutic Social Robot	US20200406468 A1	31 December 2020	Dan Stoianovici, Mohammad Mahoor	USA
27	[78]	Multi-sensor information collection analyzing system and autism children monitoring auxiliary system	CN102176222 A	7 September 2011	Xie Lun Gong Fei Wang Zhiliang	China
28	[83]	Autism children’s complementary education robot	CN108098797 A	1 June 2018	Zhao Yunqi	China
29	[70]	A kind of intelligent robot of autism children adjuvant treatment	CN109986573 A	9 July 2019	Zhao Xuehua Li Zhao Wu Xiaodan Huang Ying Han Liping Zhang Qian Chen Huiling Li Yonghong Lu Xin	China
30	[73]	Self -closing disease children’s assistive robot and system	CN204637246 U	16 September 2016	Zheng Suiwu Yang Ailong Song Yongbo Qiao Hong Li Xiaoqing Zhao Xiang	China
31	[80]	Robot-aided system and method for diagnosis of autism spectrum disorder	US20210236032 A1	5 August 2021	Chung Hyuk PARK Hifza Javed	USA
32	[74]	Intelligent remote social adjuvant therapy robot for autism children	CN103612252 B	5 March 2014	Liu Xin Fu Dongmei Xu Junwei Xie Lun Wang Zhiliang Wu Rukun	China
33	[76]	Autism child social behavior expression characteristic analysis system based on machine learning	CN109920551	21 June 2019	Chen Dongfan Zhao Weizhi Lu Zhenyu Shen Pengcheng Zhou Qifeng Zhou Qi Liang Leilei	China
34	[81]	Emotional interaction apparatus	US20170365277 B2	21 December 2017	Chung Hyuk Park	USA
35	[87]	Collaboration between a robot and a hologram	EP3312824 A1	25 April 2018	Murdjeva Yuliana Ivanova Murdjeva Nicoletta Atanasova	Europe
36	[88]	Autism Robot with Multi-Angle Recognition Device	CN216229398 U	8 April 22	Chen Xiaoling	China
37	[79]	Automatic mobile robot for facilitating activities to improve child development	US20190270026 b2	5 September 2019	Boonserm Kaewkamnerdpong Wisanu Jutharee Settapon Santatiwongchai	USA
38	[75]	System and method of pervasive developmental disorder interventions	US20190108770 A1	11 April 2019	Gregory S. Fischer Hao Su Laurie Dickstein-Fischer Kevin Harrington Elizabeth V. Alexander	USA
39	[84]	Device and method for instilling intrinsic motivation regarding eye contact in children affected by eye contact disorders	US20170360647 B2	21 December 2017	Matthew Casey	USA
40	[77]	Autism speech feature auxiliary recognition robot and method thereof	CN112259126 A	22 January 2021	Chen Shouyan Zhang Mingyan Yang Xiaofen Zhao Zhijia Zhu Dachang	China

Lastly, assistive technology patents in the form of toys combine traditional elements and components (natural materials, textures, bubble generators, snap-in blocks, levers, joints, wheels) with computational and technological elements (LCD screens, microphones, speakers, projections, motion sensors, light and sound sensors, cameras, touch sensors, buttons). The proposals are presented in the form of objects and characters from everyday life: such as trains [89], musical instruments [90], geometric shapes, and building blocks [91,92], babies and children [93,94], balls and other sports equipment [95], puzzles [96] or animals [97]. 

The skills with which toys work are varied, such as recognition of facial expressions, compression and identification of emotions [89,90,95] communication and language [93,98,99], imitation skills [100], understanding sequences and asking for help [97], eye-hand coordination [92,96], sensory stimulation [91], fine motor skills [94], or rehabilitation [101,102].

To meet these needs, products usually integrate different support stimuli such as musical sounds, textures, static and dynamic images, lights, fine and gross motor movements, imitation, interaction with a playmate, or interaction with the product in the form of conversation.

**Table 12:** Sample of assistive technology patents in the form of toys.

S/N	Reference	Title	Publication Number	Patent Issue Date	Patent Right Holder	Country
41	[89]	Smart robotic therapeutic learning toy	US20190184299 B2	20 June 2019	John-John Cabibihan Hifza Javed Kishor Kumar Sadasivuni Ahmed Yaser Alhaddad	USA
42	[91]	Sensory engagement toy	US20220054796 A1	24 February 2022	Trude McGreevy	USA
43	[90]	System and method for associating auditory stimuli with visual depictions	US20140045158 A1	13 February 2014	Movsas Tammy Zietchick	USA
44	[97]	Method and apparatus for developing a person’s behavior	US20070117073 B2	24 May 2007	Walker Michele A. Walker Jeffrey M. Reilly Daniel J.	USA
45	[99]	Intelligent dialogue psychotherapy device for infantile patient with autism	CN214232379 U	21 September 2021	Yao Li	China
46	[93]	Social game teaching aid for children with autism spectrum	CN211827784 U	30 October 2020	Fan Rongxiu	China
47	[101]	Rehabilitation exercise toy for children suffering from infantile autism	CN106139577 A	23 November 2016	Shi Yuanwu Chen Wang Chu Xuejing	China
48	[92]	Autistic child toy	CN211585212 U	29 September 2020	He Jinghao Li Nan Li Qiang Sun Jianhua Wang Pei Yang Zhimei	China
49	[98]	Autistic children language communication training system, toy and device	CN208839045 U	10 May 2019	Li Jing Ouyang Ziwei	China
50	[100]	Autistic child treating toy	CN204364892 U	3 June 2015	He Lina	China
51	[95]	Childs ball toy with changing facial expressions and features	WO2019180397	26 September 2019	James Martin	World
52	[96]	A toy for children diagnosed with autism spectrum and cerebral palsy	AU2013903846 A	24 October 2013	Daniel Javier Da Silva	Australia
53	[103]	Picture arragement teaching aid	CN207249926 U	17 April 2018	Liu Yanhong Hu Xiaoyi Fan Tianrun Huang Weixin Bi Jianming	China
54	[102]	Rehabilitation zoomorphic robot similar to a plush toy, dedicated to work with children with autism spectrum disorders	PL433091 A1	6 September 2021	Konrad Niderla Marcin Maciejewski	Poland
55	[94]	Building blocks subassembly	CN206995863 U	13 February 2018	Liu Yanhong Hu Xiaoyi Fan Tianrun Huang Weixin Bi Jianming	China

### 3.3. Analysis of Companies and Commercial Solutions

Analysis of the supply-demand relationship of TA products for ASD showed that there are few companies dedicated to the design, manufacture, and marketing of these kinds of products, with the “low tech” and “mid-tech” being the most frequent scopes and “high tech” products being less common in the portfolios of the different organizations. Most of the companies belong to the toy sector. It should be noted that of the selected companies, 61% of the organizations found are developers and manufacturers, the rest being distributors and marketers. As for the headquarters, most of the manufacturers are located in the USA. However, they are harder to find in European or Asian companies. For this analysis, a sample was selected with a total of 18 companies with the following distribution: 5 manufacturing companies, 6 manufacturers and distributors, and 7 distributors. The selection was carried out according to the following criteria: the variety of products offered, the scope of distribution, and the adaptability of the products to the specific skills of the ASD.

A selection of companies dedicated to the mid/high tech assistive technology sector for children with ASD was made. Of the 6 selected companies, 2 are dedicated to the creation of virtual content, 2 to the development of assistive robots, and another 2 to the development of interactive and intelligent products of medium-high assistive technology. Additionally, of the 6 companies selected, 5 are developers, manufacturers, and producers of such products.

Many companies dedicated to assistive technology for children with ASD belong to the toys sector, since toys are essential tools for the motor, cognitive, and social development of these children. Thus, there are several companies dedicated to the development, manufacture, and distribution of these toys. A selection of 12 toy companies was made; however, only 6 of them are manufacturers of these products, and the rest of the companies are only dedicated to sale and distribution. Therefore, it was concluded that there are very few companies dedicated to the development and manufacture of toys for children with ASD.

Similarly, manufacturing companies were classified into two groups according to their scope: (1) the toy sector and (2) the medium-high technology TA sector. First, this analysis focused on the toy sector. In general, the product portfolios of the toy sector have a wider variety; they are structured according to (1) target audience (type of disability, age), (2) type of product (educational, playful, furniture, etc.), and (3) needs and skills to be developed. In this last group, 62 different categories of toys were found. Table 13 shows the 7 most common categories among toy companies. As can be seen, “language and communication” and “social” skills, both related to collaboration, are among the most common within companies’ product portfolios.

The number of products offered by the companies presented in this study is 112,234. After an analysis of these products, a classification was made in the 7 most common categories presented in Table 11. This classification of products is shown in Table 14. In this table, it can be observed that “social” and “language and communication” skills, both related to collaboration, are again among the two categories with the highest offer of products. 

**Figure 9:**
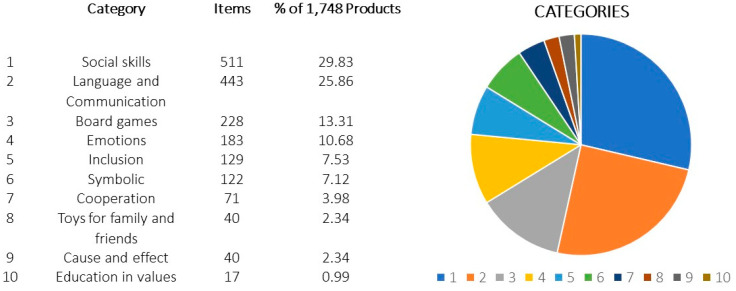
Categories of toys related to collaboration.

Analyzing products dedicated to the use of strategies and collaborative actions for the development of skills, the offer of products amounts to 1784. These were classified into the subgroups of Figure 9, where the parameters “number of items” and “participation by category” are shown in %. As can be seen, the categories of social skills’, “Language and communication”, “Board games” or “emotions” have a wide variety of products compared to the categories of “cooperation”, “Toys for family and friends”, “cause and effect”, and “education in values” with a very scarce offer. The results show a deficiency of products dedicated to the development of collaboration and cooperation, especially games and toys that allow the participation of several users simultaneously. This situation is a niche and a market opportunity, given the importance of collaborative and cooperative play in the development of skills, which has been widely demonstrated [3,4,6,9,10].

At this point, the AT products are analyzed. Below is a selection of 27 assistive technology products dedicated to improving the social and collaborative skills of children with ASD. These products were divided into three groups: Virtual Environments (Table 15), Robots (Table 16), and Toys (Table 17); these products are a representative sample of the global offer available in the market. 

**Figure 10:**
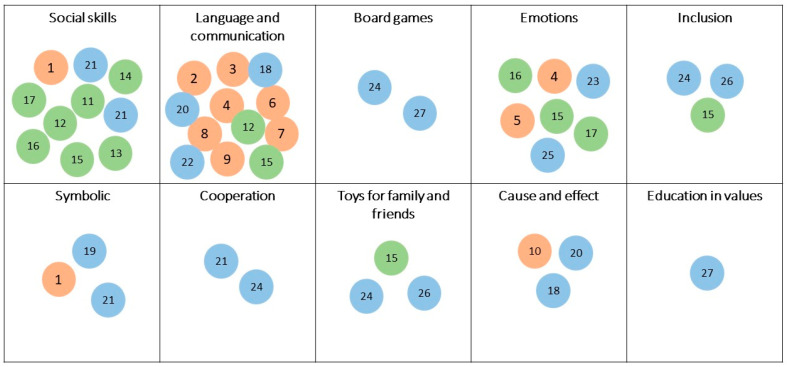
Analysis of the sample based on the categories of toys intended for collaboration.

These products were analyzed based on the categories of social skills identified in Figure 10. The results are shown in Figure 10; the different colored circles differentiate the types of products: orange “applications and virtual environments”, green “robots”, and blue “toys”. The numbers correspond to the ones on Table 15, Table 16 and Table 17.

These results are consistent with those shown in Table 13 and Table 14. The categories “Social Skills” and “Language and Communication” are the most offered, followed by “emotions”. The rest have a very reduced offer. The results show a low availability of assistive technology products in relevant categories such as “Cooperation”, “Board Games”, or “Education in Values”, which require multi-user interfaces for their use. 

From the analysis of Table 14, Table 15 and Table 16, it can be concluded that technology related to automation, self-adaptation to the environment, intelligent behavior, autonomy in interaction, and multifunctionality significantly increases the cost of products. It should be noted that the price of the most advanced robots amounts to 21,000 euros; they are usually intended for professional therapy settings and are not affordable for domestic contexts. The toys available for families do not incorporate the technological level; nor do they follow the trends of products intended for typically developing children. The situation described generates a negative impact on this group. The lack of availability and access to this type of product reduces the possibility of working more efficiently on some needs related to social skills and communication; instead, families should use products with similar functionality but designed with virtual interfaces; generally, these are available through mobile applications, for which there is a greater offer at more affordable prices. However, these lack tangibility and materiality, aspects especially important for the usability specialized in ASD [37]. The inclusion of tangible elements makes an interface more intuitive, in addition to providing tactile stimulation to the user, a key requirement to increase children’s interest in a product. 

Lastly, the results show a lack of medium-high-technology assistance products dedicated to the development of social and collaborative skills, especially in the categories “Cooperation”, “Board Games” and “Education in values”; likewise, the analysis reveals an insufficiency in the availability of products with an adequate quality-price ratio to familiar environments. This situation is a niche and market opportunity for developers and manufacturers. 

### 3.4. Technological Trends in Assistive Technology for Children with ASD

Within the field of products for autism spectrum disorder, one of the main research areas is focused on the development of assistive technology. The International Classification of Functioning, Disability, and Health (CIF, WHO, Geneva, Switzerland) defines assistive products and technology as “any product, instrument, equipment, or technology adapted or specially designed to improve the functioning of a person with a disability” [132]. Assistive technology tools have a significant impact on the well-being of children with autism spectrum disorder, providing new opportunities for therapy methods that are meant to improve different skills that contribute to the integration of these children into society. This assistive technology differentiates between:

- Low-tech: traditional tools and methods that use non-interactive products or do not use energy).
- Mid-tech: include simple electronic elements, such as recorders, e-books, headphones, and visual timers.
- High-tech: include electronic and computerized elements that improve efficiency, speed, and accessibility.

Low-tech tools used in language therapies are based on alternative and augmentative communication systems (AAC). They use symbols or images to facilitate communication and expression. The Picture Exchange Communication System (PECS) is one of the most common approaches, helping users communicate their needs and preferences through images [133]. Users usually carry these images and symbols in a book. Despite the benefits related to manufacturing cost, ease of use, or environmental impact, low-tech tools are not flexible and do not adapt to the evolution of user needs. For this purpose, mid-tech [134]) and high-tech products integrate interactive and smart functions with multisensory reinforcements, making the user experience more intuitive, adaptable, and dynamic. Furthermore, the health sector is significantly influenced by the concept of “Industry 4.0”, taking advantage of enabling technologies such as process automation, digitalization, and emerging information and communication technologies (ICTs) that have been powered by the Internet of Things (IoT), obtaining cyberphysical systems and smart products [135].

In addition to having an immediate positive influence on communication abilities, assistive technology can help children with ASD enhance their social skills [24]. These instruments are predictable, and the repetitive nature gives the child a better sense of security [136]. Since they can offer continuous feedback, they are also simpler to comprehend. To address the current social challenges, the concept of “sensing, smart, and sustainable (S3)” originated in this setting [137]. The ability of a product to recognize changes in the environment is referred to as the “sensing concept”. The term “smart concept” describes a product’s ability to combine control and actuation functions in order to understand situations and take decisions in a predictive or adaptive way. Finally, the “sustainable concept” is employed to create sustainable products while taking economic, social, and environmental design objectives into consideration.

The interactive properties of these products allow for different modes of interaction. Some of the most common ones in the literature are:

- Dialogue interaction: users interpret interaction as a conversation in which the various components are employed to convey information and await a response. [138].
- System interaction: the method by which the product gathers data and interprets it in order to later mold and determine how the conversation will proceed [139].
- Tool Interaction: users are in control of the product and use it for a certain purpose. The initiative is on the user’s side [139].
- Media interaction: the product mediates communication between people [139].
- Transmission interaction: the product presents information to the user for him/her to learn and interpret [138]).
- Implicit interaction: the product reacts to human or non-human activity state [138].
- Agent-based interaction: how the user-product interaction develops is determined by the user’s instructions to carry out a particular task [37].

These interactive qualities are linked together. When they are combined, the interaction can be adjusted to the child’s development, leading to an evolution of dialogue. 

These products may also be classified as smart products depending on their features. Smart toys “are new forms of toys that incorporate tangible objects and electronic chips to provide two-way interactions that lead to purposeful tasks with behavioral or cognitive merit” [140]. The seven characteristics of a smart product, according to Rijsdijk and Hultink, are personality, human contact, capacity of cooperation, multifunctionality, reactivity, adaptability, and autonomy [141]. In this way, these characteristics are used to evaluate how smart a product is.

Therefore, in order to design the interactive and smart qualities of products, two domains must be taken into account [135]:

- Sensing domain: sensors selection, electronic and electrical design, and mechanical design, in order to design a sensing system.
- Smart domain: design of the smart functions and components, as well as connectivity system, and integration with the physical components.

For the above reasons, some of the most important topics, research lines, and opportunity areas regarding technology and the treatment and well-being of people with autism spectrum disorder include: Robotics, Digital imaging processing, wearable systems, Internet of Things, Telemedicine, Digital medicine, and mobile apps, Communication networks, Monitoring systems, Cognitive computing, Machine learning, Big Data, Analytics technics, Human-machine and AI interfaces, and Augmented and Virtual Reality [142].

## 4. Discussion

The results of the state-of-the-art analysis show the reduced availability of products that act as facilitators of the special needs of children with ASD. Similarly, the unbalanced relationship between supply and demand for these devices on the market means that parents, guardians, and other personnel involved in therapy and education must choose to use unadapted products or self-manufacture them. 

Among the 248 resources found focused on autism, 122 were identified as dedicated to collaboration and social skills, most of them being case studies (34.5%) focused on the development and validation of techniques and tools for the work of these skills. Similarly, there is a consolidated line of interdisciplinary research on collaborative design and co-design between the different agents involved in the vital context of children with ASD (teachers, parents, therapists, educators, psychologists, and health professionals). However, there is a clear shortage of design methods and tools dedicated to the development of assistive technology that improves the collaboration and social skills of children with ASD, being more frequent in the low-tech category, and less for mid/high-tech. 

It should be noted that the scientific community is concentrating great efforts on demonstrating the benefits of mid/high-tech assistive technology. Most resources are allocated to the development of applications or virtual environments, especially those focused on improving collaboration and social skills. These types of proposal can be very beneficial, as they offer great flexibility in content and personalization. Different studies have shown the potential of these applications in the therapy of children with ASD [2,4,6,14,15,16,17,33,34,35,36]. However, most of these resources remove tangibility and materiality features from the design, especially important aspects that make the interface more intuitive, offer better tactile stimulation for users with ASD, and increase the interest of children in the product [37]. Similarly, there are a greater number of patents dedicated to virtual environments and applications compared to interactive toys or robots focused on social and collaborative skills. Most of these patents focus on the recognition of emotions or communication and language; however, a lack of proposals of products that improve cooperation skills (collaboration of several users simultaneously) was identified. 

In the commercial context, there is a large number of companies dedicated to products for children with ASD. Of the sample analyzed in this work, 61% correspond to manufacturers and developers, and 49% are dedicated to sales and distribution. However, specifically for the toy companies, only 50% are manufacturers, the rest being distributors. That is, there are few companies dedicated to the development of toys for children with ASD, and the solutions proposed are mainly from research centers. Within the product portfolios of these companies, the most offered product categories are related to the development of “social skills” and “language and communication” (both related to collaboration). However, the available products lack multi-user interfaces, which prevents these skills from working optimally, as it does not allow the participation of several users simultaneously. This situation is a niche and market opportunity, given the importance of collaborative and cooperative play in the development of other skills. It should be noted that the value for money of high technology, specifically in the case of therapy robots, makes the cost of this type of product very high. This situation causes these devices to be destined for therapy and professional settings, as they are not affordable for most families. In contrast, products in the low- and mid-tech categories do not integrate technology and trends similar to those currently available in toys marketed for typically developing children; this situation causes inequality for this group. To solve the above inconveniences, families choose to use mobile applications, with a higher offer and lower prices. The results show the existence of current unmet demand for medium-high assistive technology products, specialized in social and collaborative skills, specifically in the categories of “Cooperation”, “Board Games”, and “Education in Values”.

As a final contribution and based on the results of the review, a proposal of the most relevant lines of work that can contribute to knowledge related to the design of interactive and intelligent products for children with ASD is offered:

- Improvement and proposal of new methods and design tools for assistive technology products that are correctly adapted to the user with ASD and aimed at improving collaboration and social skills.
- Identification of the needs and technological preferences of children with autism, as well as the adaptations (physical, cognitive, and sensory) of mid- and high-tech AT products according to the signs and symptoms of the condition. Integrate in an equitable way the technological development trends available in the market for users with typical development, to end the technological gap of the ASD collective.
- Study of the optimal “aesthetic-functional” requirements for AT for autism. Current digitalization trends must properly integrate the balance between tangibility and virtuality, combining traditional strategies, methods, and materials with new technologies. This balance will allow the user to have more comfortable, flexible, and adaptable products for the user with ASD.
- The development of assistive technology meant to improve collaboration and social skills through multi-user interfaces, which guide the game to cooperation and education in values. These can be beneficial for the integration of this group into the society from an early age.

## 5. Conclusions

This work carried out an exhaustive analysis of the scientific literature, as well as market research and trends, in order to explore the state-of-the-art of assistive technology and interactive and smart products for children with ASD, specifically those aimed at improving social and communication skills. This work identifies the need for therapies to include correctly adapted products that facilitate positive interdependence between the child with ASD and his playmate. The results show an unbalanced relationship between the supply and demand for these devices in the market. They also identified a clear lack of design methods and tools dedicated to the development of AT products that improve the collaboration and social skills of children with ASD. The scientific community is concentrating great efforts on demonstrating the benefits of mid/high-tech assistive technology. However, most of the resources are allocated to the development of applications or virtual environments, which eliminates from the design of the devices the characteristics of tangibility and materiality; these aspects are especially important for the intuitiveness and stimulation of users with ASD. It also follows in both the commercial sector and the field of patents that most products and inventions focus on the recognition of emotions or communication and language, but there is a lack of products dedicated to the development of cooperation and education in values for children with ASD. It should be noted that given the importance of collaborative play for the development of personal and social skills, it would be interesting to focus research on the field of interactive and smart toys, as these types of products play an important role in the development of the collaboration skills of these children. Especially, products that allow the participation of several users simultaneously through multi-user interfaces are required. Focusing efforts and research resources on these types of devices will facilitate the integration of these people into society, avoiding social rejection, and favoring a better quality of life through inclusive design. 

## Figures and Tables

**Table 1:** Types of sources.

Source Type	Record Count	% of 328
Article	213	64.94
Book/Book Section	33	10.06
Conference	55	16.77
Thesis	10	3.05
Generic	17	5.18

**Table 2:** Bibliography categorization.

	Category	Record Count	% of 213	Dates
1	Article Reviews	16	4.88	2016–2021
2	Autism (basis)	21	6.40	2004–2022
3	Autism (Models and Therapies)	26	7.93	2000–2021
4	Techniques and tools (basis)	60	18.29	2001–2021
5	Techniques and tools (case studies)	57	17.38	2005–2021
6	Design Methodologies and Guidelines	83	25.30	2001–2021
7	Play and Toys	26	7.93	2000–2021
8	Interactive and Smart Design	17	5.18	2004–2020
9	COVID19 and Autism	22	6.71	2020–2021

**Table 3:** Main journals of bibliographic articles.

	Source Title	Record Count	% of 204
1	*Journal of Autism and Developmental Disorders*	17	7.98
2	*International Journal of Social Robotics*	7	3.29
3	*Research in Autism Spectrum Disorders*	6	2.82
4	*Computers in Human Behavior*	4	1.88
5	*Research in Developmental Disabilities*	4	1.88
6	*Autism*	4	1.88
7	*Sensors*	4	1.88
8	*ACM Transactions on Accessible Computing*	3	1.41
9	*Autism Research*	3	1.41

**Table 4:** Countries represented in affiliations in the bibliographic study.

Countries/Region	Record Count	% of 328
United States of America	103	31.40
United Kingdom	40	12.20
Spain	32	9.76
Italy	21	6.40
Australia	11	3.35
Canada	11	3.35
Denmark	10	3.05
China	10	3.05
Greece	9	2.74
The Netherlands	9	2.74

**Table 5:** Patent search.

Virtual Environments	
Search Details	Results
Social Platform Autism	3
Platform Collaboration Autism	0
Platform therapy autism	1
Platform emotion autism	0
Platform cooperation autism	0
Platform speech autism	1
Social virtual autism	7
Virtual collaboration autism	0
Virtual therapy autism	8
Virtual Emotion autism	1
Virtual cooperation autism	1
Virtual speech autism	1
Robots	
Social Robot Autism	4
Robot Collaboration Autism	2
Robot therapy autism	4
Robot emotion autism	4
Robot cooperation autism	0
Robot speech autism	3
Robot autism	24
Toys	
Toy collaboration autism	0
Social toy autism	1
Toy therapy autism	0
Toy emotion autism	0
Toy cooperation autism	0
Cooperative play autism	0
Toy speech autism	0
Sensory toy autism	1
Toy autism	17
Collaboration toy	7

**Table 6:** Most common JCR areas in the bibliographic study.

	Category	Record Count	% of 213
1	PSYCHOLOGY, DEVELOPMENTAL—SSCI	38	17.84
2	REHABILITATION—SSCI	25	11.74
3	EDUCATION, SPECIAL—SSCI	18	8.45
4	EDUCATION & EDUCATIONAL RESEARCH—SSCI	14	6.57
5	NEUROSCIENCES—SCIE	10	4.69
6	ROBOTICS—SCIE	10	4.69
7	PSYCHIATRY—SSCI	9	4.23
8	PSYCHOLOGY, EXPERIMENTAL—SSCI	8	3.76
9	PEDIATRICS—SCIE	7	3.29
10	REHABILITATION—SCIE	7	3.29
11	PSYCHOLOGY, MULTIDISCIPLINARY—SSCI	7	3.29

**Table 7:** Resources focused on autism for each category.

	Category	Record Count	% of 248
1	Article Reviews	15	6.05
2	Autism (basis)	21	8.47
3	Autism (Models and Therapies)	26	10.48
4	Techniques and tools (basis)	44	17.74
5	Techniques and tools (case studies)	57	22.98
5	Design Methodologies and Guidelines	52	20.97
7	Play and Toys	26	10.48
8	Interactive and Smart Design	17	6.85
9	COVID-19 and Autism	22	8.87

**Table 8:** Resources focused on the collaboration of people with autism for each category.

	Category	Record Count	% of 122
1	Article Reviews	2	1.6
2	Autism (basis)	8	6.6
3	Autism (Models and Therapies)	11	9.0
4	Techniques and tools (basis)	22	18.0
5	Techniques and tools (case studies)	42	34.4
6	Design Methodologies and Guidelines	32	26.2
7	Play and Toys	5	4.1
8	Interactive and Smart Design	0	0.0
9	COVID-19 and Autism	0	0.0

**Table 9:** Classification of patent samples in the different sections and technological areas.

Section	Technology	Count
Electrical Engineering	Computer technology	7
	IT methods for management	1
Instruments	Control	16
	Medical technology	14
Mechanical Engineering	Handling	8
Other Fields	Furniture, Games	11

**Table 13:** Most common ASD needs-based toy categories.

Category	Sample of Products	Sample of Companies (%)
Sensoriality	10	83.4
Motricity	10	83.4
Creativity	6	50.0
Language and communication	6	50.0
Chewing	5	41.7
Social skills	4	33.4
Coordination	4	33.4

**Table 14:** Categories of Toys based on ASD needs with more offer.

Category	Count of Products	% of 112,234 Products
Sensoriality	1810	16.11
Motricity	1588	14.14
Creativity	511	4.55
Language and communication	507	4.51
Chewing	471	4.19
Social skills	443	3.94
Coordination	418	3.72

**Table 15:** Sample of commercial products in the form of virtual environments.

	Product	Reference	Description	Price
1	AutisMIND	[104]	Stimulates the development of Theory of Mind and Social Thinking in children with ASD.	Free
2	LetMeTalk	[105]	Allows children to express sentences with images.	Free
3	HelpMeTalk	[106]	Includes support in several languages, animation (GIF), and effective exercises to learn to speak.	APP purchases: 5–245 €
4	Aut2Talk:	[107]	Keyboard for people with autism. Includes names, feelings, needs, pronouns, and word endings.	Free
5	AutismXpress	[108]	Helps people with autism recognize and express emotions with facial expressions. There are 12 buttons with cartoons representing emotions.	Free
6	CommBoards	[109]	Based on the Picture Exchange Communication System (PECS).	20 €
7	Leeloo:	[110]	Uses a wide range of image options to help nonverbal children with autism communicate with their caregivers. It is based on the Picture Exchange Communication System (PECS) and includes the principles of augmentative and alternative communication (AAC).	APP purchases: 8–140 €
8	ECO: Easy Communicator	[111]	ECO is an augmentative and alternative communication system (AACS) that, through images, videos, audio, and texts, provides help to people with difficulties in communicating. It has been designed and validated in close collaboration with real entities and users.	Free
9	Injini	[112]	It is stimulating and feels like a fun game, promoting the development of fine motor and language skills, as well as spatial awareness, memory and visual processing, and understanding of cause and effect.	30 €
10	Proloquo2Go	[113]	Communication app for people who cannot speak or need help being understood. Features natural sounding voices, including real children’s voices; AAC (augmentative and alternative communication) tool.	120 €

**Table 16:** Sample of commercial products in the form of robots.

	Product	Reference	Description	Price
11	Nuka	[114]	With the appearance of a stuffed seal, it offers companionship and assistance to people with special needs. It can replace animal therapies as it is designed to interact with humans.	4500 €
12	Leka	[115]	Includes visual and auditory support in the form of conversation, music, lights, and colors; it can move. It integrates different game modes.	650 €
13	NAO	[116]	Facilitates therapy for children with neurodevelopmental disorders. This anthropomorphic robot is used as a tool to generate human-robot interactions in children with ASD.	8000–21,000 €
14	Kaspar	[117]	Social robot the size of a child that helps children with autism improve their ability to interact. It is capable of performing basic movements and expressions.	2000 €
15	QTRobot	[118]	Expressive social robot for parents of children with autism for at-home education and teaching social, emotional, and communication skills.	2065 €+ Software (148 €/month)
16	Pepper	[119]	It is able to recognize faces and basic human emotions. Engages with people through conversation and a touch screen.	13,000–13,500 €
17	Keepon	[120]	Toy version of Keepon Pro, developed in partnership with UK-based Wow! Stuff. In its touch mode, it responds to pokes, pats, and tickles with a wide variety of emotional movements and sounds. In its dance mode, it hears the beat of music or clapping and dances in synchronized rhythm.	40 €

**Table 17:** Sample of commercial products in the form of toys.

	Product	Reference	Description	Price
18	Resonance microphones	[121]	The voice is amplified through the soundboard. It promotes sensory development by stimulating speech, language, and curiosity.	11 €
19	Visual sequences of tasks	[122]	It shows the sequences of an action to be put in order. Working on the decomposition of events into actions, we manipulate more abstract concepts such as the past, present, and future.	32 €
20	Repeating toys	[123,124]	A toy that repeats everything the child says. It encourages children to work on the articulation of words, the time, and the volume to which we speak.	25 €
21	Tell me what you see	[125]	It includes a book with pictures and a set of figures. The player must describe what he sees in the picture. The other must listen carefully and reproduce what is being described with the figures.	90 €
22	Go Talk express 32	[126]	Includes a panel with images. The user can select images to communicate. It is intended for nonverbal children.	573.45 €
23	Emoti-capsules	[127]	Playful support to develop emotional intelligence. Each capsule represents an emotion. Children hide a drawing, or a photo, of what inspires that emotion.	20 €
24	The wheel of self-esteem	[128]	The wheels indicate verbal or non-verbal actions that must be carried out in the form of concrete photographs. Actions may involve the collaboration of a partner.	19.90 €
25	The color monsters	[129]	Helps children with ASD understand and differentiate different emotions through colors.	42.50 €
26	Table of light	[130]	It is a large table that allows you to paint on it. It has lights that make the paintings look neon. Allows children to collaborate on the same table.	90 €
27	Cooperative puzzles	[131]	Cooperative puzzle for different players to complete turn-based puzzles.	20.50 €
